# Determination of Germination Response to Temperature and Water Potential for a Wide Range of Cover Crop Species and Related Functional Groups

**DOI:** 10.1371/journal.pone.0161185

**Published:** 2016-08-17

**Authors:** Hélène Tribouillois, Carolyne Dürr, Didier Demilly, Marie-Hélène Wagner, Eric Justes

**Affiliations:** 1 INRA, UMR AGIR 1248, 24 chemin de Borderouge–Auzeville, 31320, Castanet-Tolosan, France; 2 INRA, IRHS 1345, 42 rue George Morel, 49071, Beaucouzé, France; 3 GEVES, Station Nationale d’Essais de Semences, 25 rue George Morel, 49071, Beaucouzé, France; UC Davis MIND Institute, UNITED STATES

## Abstract

A wide range of species can be sown as cover crops during fallow periods to provide various ecosystem services. Plant establishment is a key stage, especially when sowing occurs in summer with high soil temperatures and low water availability. The aim of this study was to determine the response of germination to temperature and water potential for diverse cover crop species. Based on these characteristics, we developed contrasting functional groups that group species with the same germination ability, which may be useful to adapt species choice to climatic sowing conditions. Germination of 36 different species from six botanical families was measured in the laboratory at eight temperatures ranging from 4.5–43°C and at four water potentials. Final germination percentages, germination rate, cardinal temperatures, base temperature and base water potential were calculated for each species. Optimal temperatures varied from 21.3–37.2°C, maximum temperatures at which the species could germinate varied from 27.7–43.0°C and base water potentials varied from -0.1 to -2.6 MPa. Most cover crops were adapted to summer sowing with a relatively high mean optimal temperature for germination, but some Fabaceae species were more sensitive to high temperatures. Species mainly from Poaceae and Brassicaceae were the most resistant to water deficit and germinated under a low base water potential. Species were classified, independent of family, according to their ability to germinate under a range of temperatures and according to their base water potential in order to group species by functional germination groups. These groups may help in choosing the most adapted cover crop species to sow based on climatic conditions in order to favor plant establishment and the services provided by cover crops during fallow periods. Our data can also be useful as germination parameters in crop models to simulate the emergence of cover crops under different pedoclimatic conditions and crop management practices.

## Introduction

Diverse cover crop species are increasingly used in agriculture during fallow periods to provide various ecosystem services, such as catching nitrate to limit water pollution, increasing nitrogen available for the next crop and protecting soils against erosion [1–3]. These species can be sown as a sole crop (monoculture) or intercropped (species mixture) to provide the services [4,5]. In temperate European climates, cover crops are usually sown in late summer for two to several months according to the fallow-period duration determined by the cash-crop succession. One challenge is to successfully establish a cover crop stand in late summer to obtain all the expected services within a few months. Seed germination and seedling emergence are crucial processes that depend on climatic conditions and are the first key steps for plant establishment. Temperature and water potential are particularly important factors that regulate germination [6,7]. In summer, high temperatures and low water availability can frequently occur, which influence the ability of cover crops to germinate in the field [8,9]. The diversity of climates and sowing dates requires choosing the best cover crop species to sow, which requires better knowledge about germination response to temperatures and water potentials for a large number of species among different families (Brassicaceae, Poaceae, Fabaceae, etc.). If the species cannot germinate because of too high temperature and too low water potential in the seedbed, the plant cover will never be correctly established.

Seed germination is widely examined through the final germination percentage and germination rate, defined as 1/time to germinate a defined seed percentage [10]. The range of temperatures favorable for seed germination can be described by cardinal (minimum, optimum and maximum) temperatures [11]. The minimum temperature (T_0_) is the lowest temperature at which a seed can germinate. The optimum temperature (T_opt_) is the temperature at which the germination rate is highest, and the maximum temperature (T_max_) is the highest temperature at which a seed can germinate. The response of germination rate to temperature can be fitted to the Yin model [12], in which three parameters are the cardinal temperatures. Base temperature (T_b_) value is similar to the T_0_ value and is used to calculate cumulative thermal time. Base water potential (Ψ_b_) is also a germination parameter used to represent species response to soil water availability. Ψ_b_ is the lowest water potential at which a seed can germinate. Calculating these germination indicators allows the germination ability of species to be compared under various temperatures and water potentials. They can also be useful for modeling species germination and emergence under contrasting climatic conditions; these parameters are more and more often available in the literature [13].

The influence of temperature and water availability on germination have been studied for many species, but only a few studies investigated the germination of cover crop species such as white mustard, Italian ryegrass or hairy vetch [2,14–17]. No comparative study exists that considers a range of temperatures and water potentials for seed germination with a wide range of cover crop species. Therefore, a lack of knowledge exists about cover crop germination.

The aim of this study was to determine the response of germination to temperature and water potential and to calculate cardinal temperatures, T_b_ and Ψ_b_, for diverse cover crop species from several families. These characteristics were then used to identify functional groups of cover crop species with the same germination response to climatic conditions to help choose the species most adapted to summer sowing.

## Materials and Methods

### Cover crop species and seeds

Thirty-six taxa (34 species and two varieties of *Vicia faba* and *Pisum sativum*) from six botanical families were selected to ensure a wide range of species used as cover crops in Europe (Table 1). All species had C_3_ photosynthetic pathways, except *Setaria italica* and *Sorghum bicolor var*. *sudanense*, which were C_4_ Poaceae. Seeds were produced and marketed by French seed companies, the mother plants were grown in optimum conditions in fields specifically managed for seed multiplication and collect. The seeds were stored only a few months. For each taxon, the same seed lot was used for all conditions tested to avoid a potential “lot effect” so as to evaluate only the influence of temperature and water potential on germination. No seed treatment was applied. To test the possible effect of the seed lot we used, even if the seeds were commercial seed lots of good quality, some experiments were replicated on a second seed lot but only for a smaller set of species. As satisfactory results were obtained we have a good confidence in the results presented in this paper for the various species analyzed.

**Table 1:** Seed mass, germination cardinal temperatures and base temperature values for the 36 cover crop species.

Family	Species	Id.	Seed weight (mg)	Minimum temperature (°C)	Maximum temperature (°C)	Optimum temperature (°C)	Base temperature (°C)
Asteraceae	*Guizotia abyssinica*	GA	3.3	8.7 ± 2.8	42.9 ± 0.3	28.7 ± 1.3	8.1 ± 0.9
	*Helianthus annuus*	HA	48.0	2.3 ± 1.1	36.0 ± 0.0	32.5 ± 0.6	4.4 ± 0.5
		**Mean**	**25.7 ± 31.6**	**5.5 ± 4.5**	**39.5 ± 4.9**	**30.6 ± 2.7**	**6.3 ± 2.6**
Brassicaceae	*Brassica carinata*	BC	5.0	0.0 ± 0.0	37.1 ± 0.2	32.3 ± 0.3	6.7 ± 0.9
	*Brassica juncea*	BJ	3.0	0.3 ± 0.6	37.8 ± 1.4	33.7 ± 0.6	6.8 ± 0.5
	*Brassica napus*	BN	2.7	0.0 ± 0.0	38.9 ± 0.5	32.7 ± 0.4	7.2 ± 0.2
	*Brassica rapa*	BR	3.7	0.0 ± 0.0	39.7 ± 0.0	33.1 ± 0.5	6.6 ± 0.0
	*Camelina sativa*	CS	1.3	0.0 ± 0.0	35.8 ± 0.4	28.3 ± 0.1	2.1 ± 0.1
	*Eruca sativa*	ES	1.3	0.8 ± 0.6	36.2 ± 0.1	32.5 ± 0.4	5.4 ± 0.1
	*Raphanus sativus*	RS	13.0	1.2 ± 0.9	39.5 ± 0.0	37.2 ± 0.2	7.3 ± 0.6
	*Sinapis alba*	SA	8.0	0.0 ± 0.0	40.4 ± 0.3	29.6 ± 0.7	1.2 ± 0.1
		**Mean**	**4.8 ± 4.0**	**0.3 ± 0.5**	**38.2 ± 1.7**	**32.4 ± 2.7**	**5.4 ± 2.4**
Fabaceae	*Lathyrus sativus*	LS	176.0	0.3 ± 0.6	39.1 ± 0.6	26.8 ± 0.7	3.5 ± 0.2
	*Lens nigricans*	LN	21.5	0.3 ± 0.5	37.4 ± 1.4	31.8 ± 2.2	0.8 ± 1.4
	*Lupinus angustifolius*	LA	179.4	1.3 ± 0.8	35.4 ± 4.2	25.7 ± 3.7	0.8 ± 0.1
	*Medicago lupulina*	ML	1.5	2.1 ± 0.5	30.3 ± 4.8	26.2 ± 4.1	0.6 ± 0.4
	*Melilotus officinalis*	MO	2.5	1.1 ± 1.1	33.5 ± 1.3	24.9 ± 2.3	0.8 ± 1.3
	*Onobrychis viciifolia*	OV	23.0	1.8 ± 1.1	31.7 ± 0.2	24.2 ± 1.2	0.0 ± 0.0
	*Pisum sativum ASSAS*	PSA	168.8	0.7 ± 0.7	33.5 ± 3.2	28.5 ± 0.9	1.1 ± 1.5
	*Pisum sativum PFX*	PSP	214.5	0.0 ± 0.0	32.0 ± 0.6	29.3 ± 1.3	7.3 ± 0.8
	*Trifolium alexandrinum*	TA	3.0	1.1 ± 1.9	41.6 ± 1.2	30.0 ± 1.4	6.1 ± 0.3
	*Trifolium incarnatum*	TI	4.7	1.5 ± 1.2	43.4 ± 0.5	26.5 ± 1.9	6.4 ± 0.3
	*Trigonella foenum-graecum*	TFG	16.0	0.0 ± 0.0	43.0 ± 0.0	30.1 ± 0.3	4.2 ± 1.0
	*Vicia benghalensis*	VB	41.4	2.6 ± 0.5	39.5 ± 0.0	23.6 ± 0.8	2.1 ± 0.1
	*Vicia faba LAURA*	VFL	442.8	0.2 ± 0.4	33.9 ± 2.7	23.8 ± 2.6	0.0 ± 0.0
	*Vicia faba SSNS*	VFS	359.6	0.5 ± 0.5	31.6 ± 0.0	28.1 ± 0.3	1.2 ± 2.0
	*Vicia sativa*	VS	53.8	0.6 ± 1.0	30 .0± 1.7	22.0 ± 0.8	4.1 ± 0.0
	*Vicia villosa*	VV	26.7	0.5 ± 0.8	33.1 ± 6.8	20.2 ± 1.1	1.4 ± 0.9
		**Mean**	**108.5 ± 137.0**	**0.9 ± 0.8**	**35.6 ± 4.5**	**26.4 ± 3.2**	**2.5 ± 2.4**
Hydrophylaceae	*Phacelia tanacetifolia*	PT	1.8	0.3 ± 0.6	27.7 ± 1.0	21.3 ± 1.0	3.6 ± 0.1
Poaceae	*Avena sativa*	AV	39.4	0.5 ± 0.8	32.7 ± 3.5	25.5 ± 4.5	2.2 ± 1.6
	*Avena strigosa*	AS	16.1	0.0 ± 0.0	35.8 ± 0.2	27.8 ± 0.4	4.8 ± 0.1
	*Lolium hybridum*	LH	3.4	0.9 ± 0.7	36.0 ± 0.0	29 ± 0.3	1.1 ± 0.3
	*Lolium multiflorum*	LM	2.7	0.4 ± 0.3	34.6 ± 1.3	30.1 ± 0.1	1.9 ± 0.3
	*Secale cereale*	SC	32.3	0.5 ± 0.7	38.1 ± 1.5	34.5 ± 1.5	0.6 ± 0.2
	*Secale multicaule*	SM	18.8	0.0 ± 0.0	37.1 ± 0.9	32.1 ± 1.1	3.1 ± 0.8
	*Setaria italica*	SI	2.2	11.3 ± 0.3	39.5 ± 0.0	36.1 ± 1.5	10.6 ± 0.1
	*Sorghum sudanense*	SS	13.8	5.3 ± 3.0	40.6 ± 1.5	35.6 ± 3.0	9.4 ± 0.6
		**Mean**	**16.1 ± 13.9**	**2.4 ± 4.0**	**36.8 ± 2.6**	**31.3 ± 3.9**	**4.2 ± 3.8**
Polygonaceae	*Polygonum Fagopyrum*	PF	25.0	3.8 ± 0.4	39.4 ± 0.2	32.3 ± 0.4	7.8 ± 0.9

Mean and standard error are calculated from four percentiles of germination percentage (20th, 30th, 40th, and 50th).

### Germination experiments

Seed germination was measured in the laboratory at eight temperatures ranging from 4.5–39.5°C, and also at 43°C for species with adequate germination at 39.5°C. Germination at temperatures ranging from 11.5–36°C was measured with an automated digital imaging system performed with a high-throughput seed germination phenotyping system, described in more details in Demilly et al [18]. This device is made of a Jacobsen table composed of grids on which flat blotter paper and seeds are placed; a continuous flow of demineralized water assures optimal moisture for seed germination. The Jacobsen table was placed in a dark room. The table is covered by a hood that automatically opened every two hours to take pictures with four cameras under white light during two minutes. Four replicates of 25 seeds were sown for each taxon in each controlled conditions, and each replicate was placed under a camera. Image analysis was performed with Fiji software [19], merging the pixels from the color images into two classes (seeds or background), and then each seed was measured by a labeling module [20–22]. A seed was considered to have germinated when its radicle pushed through the integument. Germination assessment was done by automatically counting the newly germinated seed at each step time. This method yields highly precise germination dynamics. For the extreme temperatures (4.5°C, 39.5°C and 43°C), seeds were sown on flat germination paper in plastic Petri dishes, moistened with 5 ml deionized water and placed in the dark in incubators under controlled temperatures. Deionized water was added after each measurement. Germinated seeds were counted two or three times per day depending on temperature. Seeds received light during a couple of minutes during counting. The temperature in the incubators was recorded every 30 minutes for each experiment.

For the base water potential experiment, the same numbers of seeds and replicates were used as for the temperature experiments. The seeds were sown on flat germination paper in Petri dishes to which a solution of polyethylene glycol (average mol wt 8.000 SIGMA-ALDRICH) was added to obtain a given water potential. The polyethylene glycol solutions were prepared according to Michel (1983) [23], the concentrations of PEG used are indicated below in brackets with the associated water potential. This method reproduces multiple water potentials without causing other effects on seed germination [24]. The water potentials tested were 0 MPa, -0.1 MPa (73.7 g PEG.l^-1^), -0.5 MPa (195 g PEG.l^-1^), and -0.75 MPa (250 g PEG.l^-1^) for all species. Depending on initial results, additional water potentials of -0.9 and/or -1.5 MPa (376 g PEG.l^-1^) were tested for species whose germination remained high at -0.75 MPa. For these experiments, the Petri dishes were placed in the dark in incubators at 20°C.

### Data analysis

For temperature experiments, cumulative germination dynamics over time for each species and each temperature were fitted to the Gompertz function, which expresses the cumulative percentage of germinated seeds, G_t_, at time t after sowing (h), as follows:

$$G_{t}=G_{max}.EXP\left[\left(-\frac{b}{c}\right).EXP\left(-c.t\right)\right]$$ (1)

where G_max_ is the final germination percentage (maximum cumulative germination), and b and c are shape parameters. The germination rate was calculated for G = 20^th^, 30^th^, 40^th^ and 50^th^ percentiles as 1/t(G) and fitted to the Yin model [12] for each taxon over all temperatures:

$$\frac{1}{t\left(G\right)}=EXP\left(\mu \right).{\left(T-T_{0}\right)}^{\alpha }.{\left(T_{max}-T\right)}^{\beta }$$ (2)

where T is the measured temperature, T_0_ and T_max_ are the minimum and maximum temperatures, respectively, at which germination stops, and μ, α and β are shape parameters. The optimal temperature for germination (T_opt_) was calculated for each taxon based on the parameters of Eq 2, as:

$$T_{opt}=\left(\beta .\;T_{max}+\alpha .T_{0}\right)/\left(\alpha +\beta \right)$$ (3)

T_b_ was calculated for the 20^th^, 30^th^, 40^th^ and 50^th^ percentiles based on 1/t(G), which increased linearly (1/t(G) = a×T + b) with temperature [7,25]. T_b_ was estimated as the value at which germination was null when fitting the germination rate to temperature for the linear response phase only. T_b_ was thus the mean of the four percentiles, and its standard deviation was calculated from results for all four percentiles. The same method was used to calculate Ψ_b_, but only with the 20^th^ and 30^th^ percentiles because of low germination rates (< 50%) at several water potentials.

All cardinal temperatures of germination (T_0_, T_opt_, T_max_), T_b_ and Ψ_b_ were calculated for each percentile and averaged to obtain a better estimate. All fits were adjusted by minimizing the residual sum of squares. Statistical analyses were performed using STATGRAPHICS Centurion XV software (version 15.2.06). ANOVA and a posteriori Student-Newman-Keuls tests were used with a significance level of *P*<0.05.

Hierarchical classification was performed using the Ward method based on the three cardinal temperatures and Ψ_b_. This method minimizes the total within-cluster variance. The number of final clusters was initially chosen and at each step the procedure finds the pair of cluster which minimizes the total within-cluster variance after merging [26]. We used T_0_ instead of T_b_ to estimate the minimum temperature at which the species can germinate, because T_0_ is generally estimated more precisely than T_b_. Distances between species were squared Euclidean distances.

## Results

### Temperature influence on final germination percentage and germination dynamics

**Fig 1:**
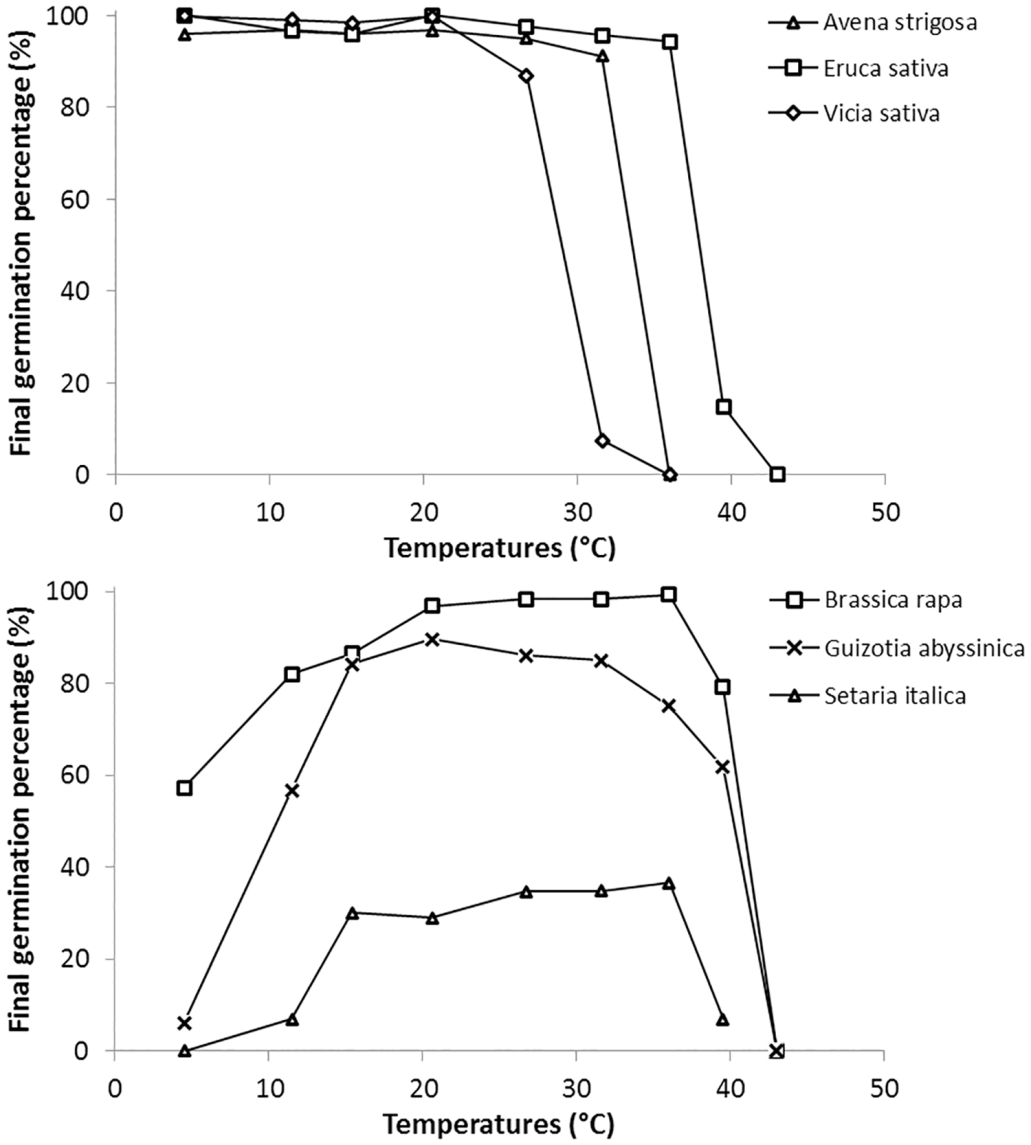
Influence of temperature on final germination percentage for six cover crop species, showing two contrasting responses (a and b).

All species reached a high maximum final germination percentage of 80–100%, except *Setaria italica* (37%), *Medicago lupulina* (62%), *Avena sativa* (66%), *Melilotus officinalis* (74%) and *Camelina sativa* (75%). Two different responses of final germination percentage to temperature were observed (Fig 1): (i) the species maintained a constant final germination percentage regardless of temperature in the range of 4.5–35°C, which sharply decreased only at 39.5°C or 43°C (Fig 1a) or (ii) the final germination percentage was low at both minimum and maximum temperatures (Fig 1b). The former occurred in particular for all Fabaceae, all C_3_ Poaceae, some Brassicaceae (*Brassica napus*, *Sinapis alba* and *Eruca sativa*), *Phacelia tanacetifolia* and *Helianthus annuus*. The latter concerned most Brassicaceae, the two C_4_ Poaceae, *Guizotia abyssinica* and *Polygonum fagopyrum*.

**Fig 2:**
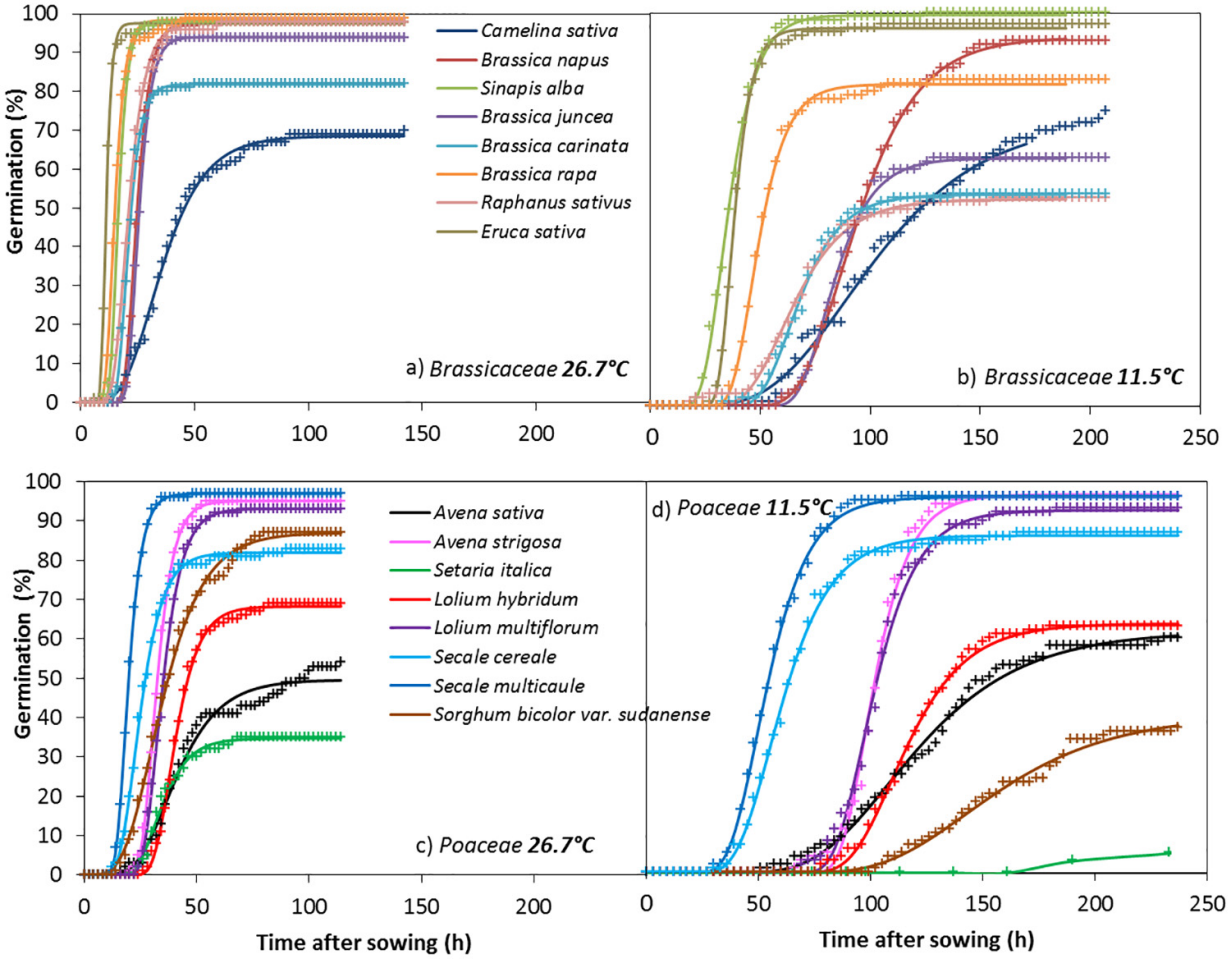
Variation in germination dynamics for Brassicaceae cover crops at a) 26.7°C and b) 11.5°C and for Poaceae cover crops at c) 26.7°C and d) 11.5°C. Crosses represent observed data and lines represent fitted Gompertz functions.

Cumulative germination dynamics varied widely among species, as illustrated for two main families: Brassicaceae and Poaceae. For Brassicaceae at 26.7°C (Fig 2a), all species began to germinate very early (8–18 h after sowing) and quickly reached a high final germination percentage (22–42 h after sowing), except *Camelina sativa*. For Poaceae at the same temperature, germination began later (12–28 h after sowing), and final germination percentages varied more among species (35–98%). At 11.5°C, germination percentage varied even more by species but remained more variable for Poaceae than for Brassicaceae. For Poaceae (Fig 2c and 2d), the largest differences between the two temperatures were observed for the two C_4_ species, *Sorghum bicolor var*. *sudanense* and *Setaria italica*, whose final germination percentages were much lower at 11.5°C, since they were reduced by 56% and 80%, respectively.

### Cardinal temperatures and base temperature

**Fig 3:**
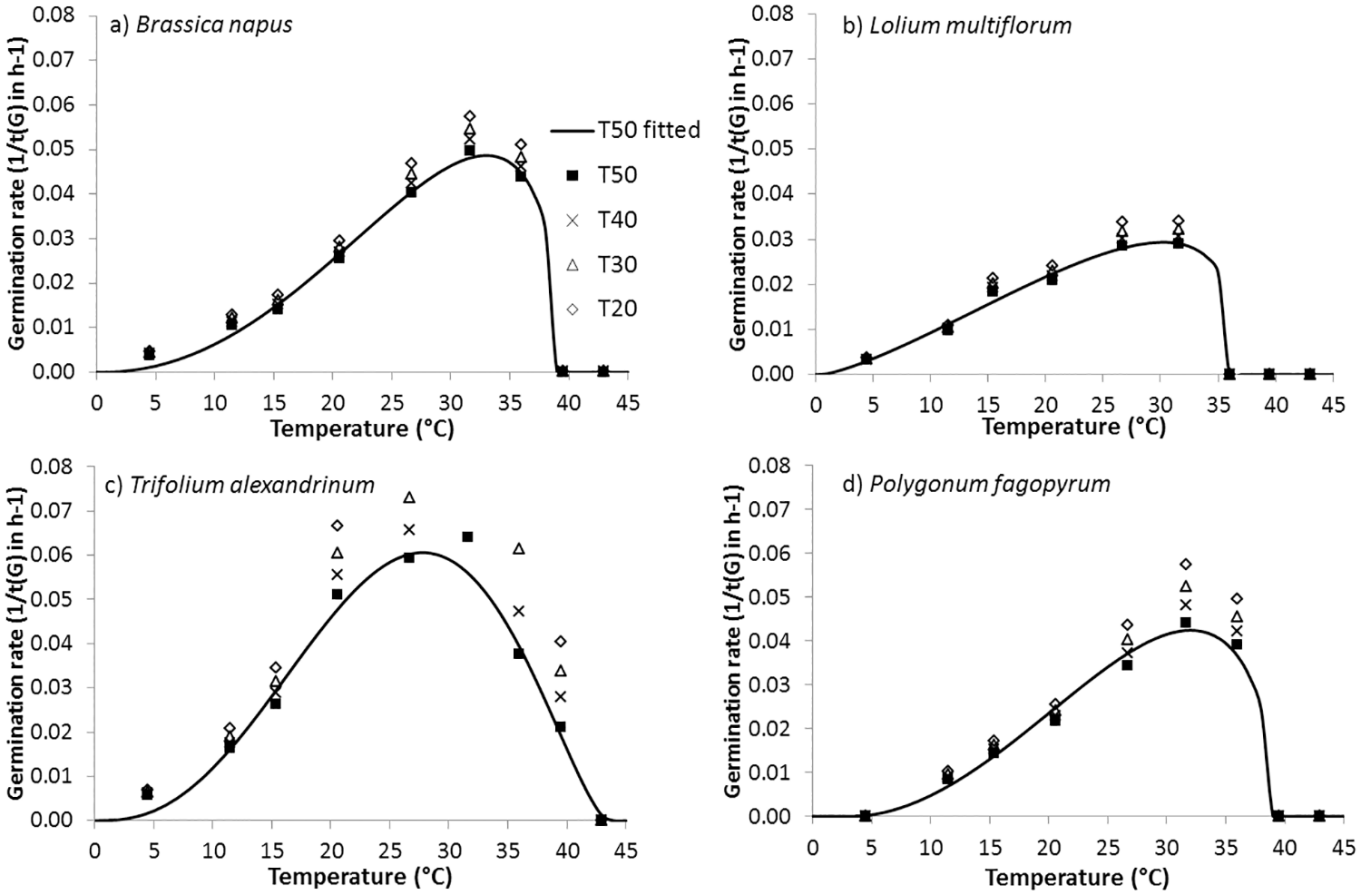
Curves fit to observed germination rates for four fractions percentages of the seed population, 20% (◊), 30% (Δ), 40% (x) and 50% (■), according to temperature, for *Brassica napus* (Brassicaceae), *Lolium multiflorum* (Poaceae), *Trifolium alexandrinum* (Fabaceae) and *Polygonum fagopyrum (*Polygonaceae*)*.

The fits of germination rate were close to the observed data, with R² from 0.81 for *Vicia villosa* to 0.99 for *Secale cereale* and *Medicago lupulina*, and a mean of 0.95 for all species (data not shown). The germination rate gradually increased from the minimum temperature to the optimal temperature and then sharply decreased (Fig 3). The different percentiles showed the same germination rate curve according to temperature, which led to similar estimates of T_0_, T_opt_ and T_max_.

Based on these fits, the mean T_opt_ of all cover crops was 29.1°C and ranged from 20.0°C for *Vicia villosa* to 37.2°C for *Raphanus sativus* (Table 1). Brassicaceae had a mean optimal germination rate higher than that of other families; it was significantly (*P* = 0.002) higher than that of Fabaceae. Among Brassicaceae, *Camelina sativa* and *Sinapis alba* had T_opt_ at significantly (*P*<0.0001) lower temperatures than other confamiliars. Among Fabaceae, T_opt_ of *Lens nigricans*, *Trigonella foenum-graecum* and *Trifolium alexandrinum* were significantly (*P*<0.0001) the highest, whereas T_opt_ of *V*. *villosa*, *V*. *sativa*, *V*. *benghalensis* and *V*. *faba* var. LAURA were the lowest. Poaceae were similar to Brassicaceae, but certain Poaceae species, such as *Avena strigosa* and *A*. *sativa*, had a T_opt_ significantly (*P*<0.0001) lower than species with the highest T_opt_, such as the two C_4_ Poaceae. Overall, mean T_0_ was 1.4°C for all cover crops and ranged from about 0.0°C for many species, especially Brassicaceae, to 11.3°C for *Setaria italica*. Mean T_max_ of all cover crops was 36.5°C and ranged from 27.7°C for *Phacelia tanacetifolia* to 43.4°C for *Trifolium incarnatum*.

**Fig 4:**
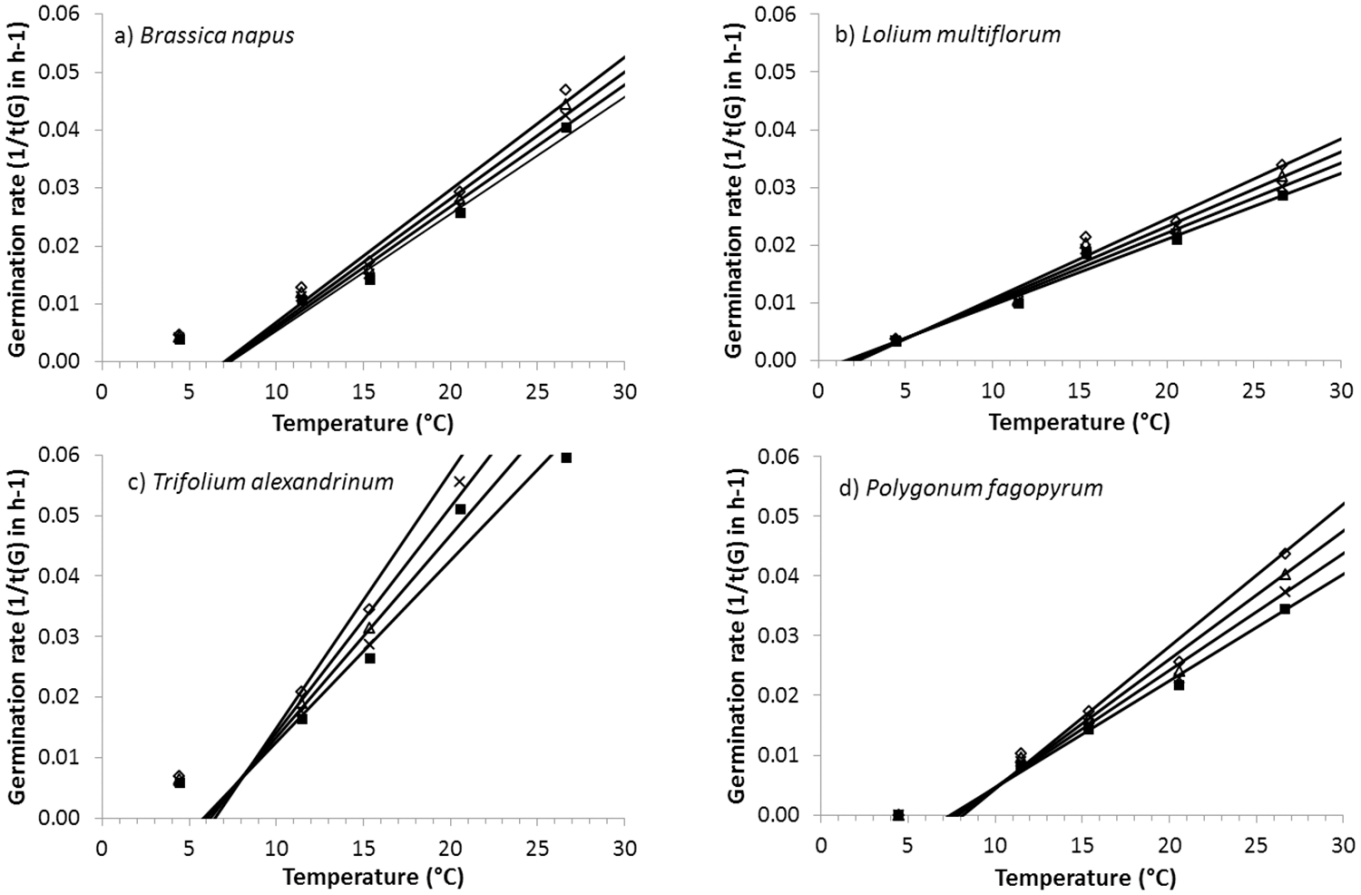
Linear relationships fit between observed germination rate and temperature for four fractions percentages of the seed population, 20% (◊), 30% (Δ), 40% (x) and 50% (■), for *Brassica napus* (Brassicaceae), *Lolium multiflorum* (Poaceae), *Vicia sativa* (Fabaceae) and *Polygonum fagopyrum* (Polygonaceae).

Germination rate usually increased linearly from 10–25°C, though minimum and maximum temperatures depended on the species (Fig 4). These fits based on the 20^th^, 30^th^, 40^th^ and 50^th^ percentiles allowed more precise determination of T_b_; differences between the T_b_ of each percentile were not significant (*P* = 0.72). For all cover crops, mean T_b_ was 3.8°C and ranged from about 0.0°C for two Fabaceae species to 10.6°C for *Setaria italica*. Overall, Brassicaceae had significantly (*P*<0.05) higher mean T_b_ (5.4°C) than Fabaceae (2.5°C). Among Fabaceae, the two *Trifolium* had significantly (*P*<0.0001) higher T_b_ (mean = 6.3°C) than other confamiliars. Two contrasting behaviors occurred among Poaceae: the two C_4_ species had a high mean T_b_ (10.0°C), while the C_3_ species had a mean T_b_ (2.3°C) similar to that of Fabaceae. Finally, T_b_ was positively and significantly (*P*<0.05) correlated with the three cardinal temperatures but with low R² (0.30).

### Base water potential for germination

**Fig 5:**
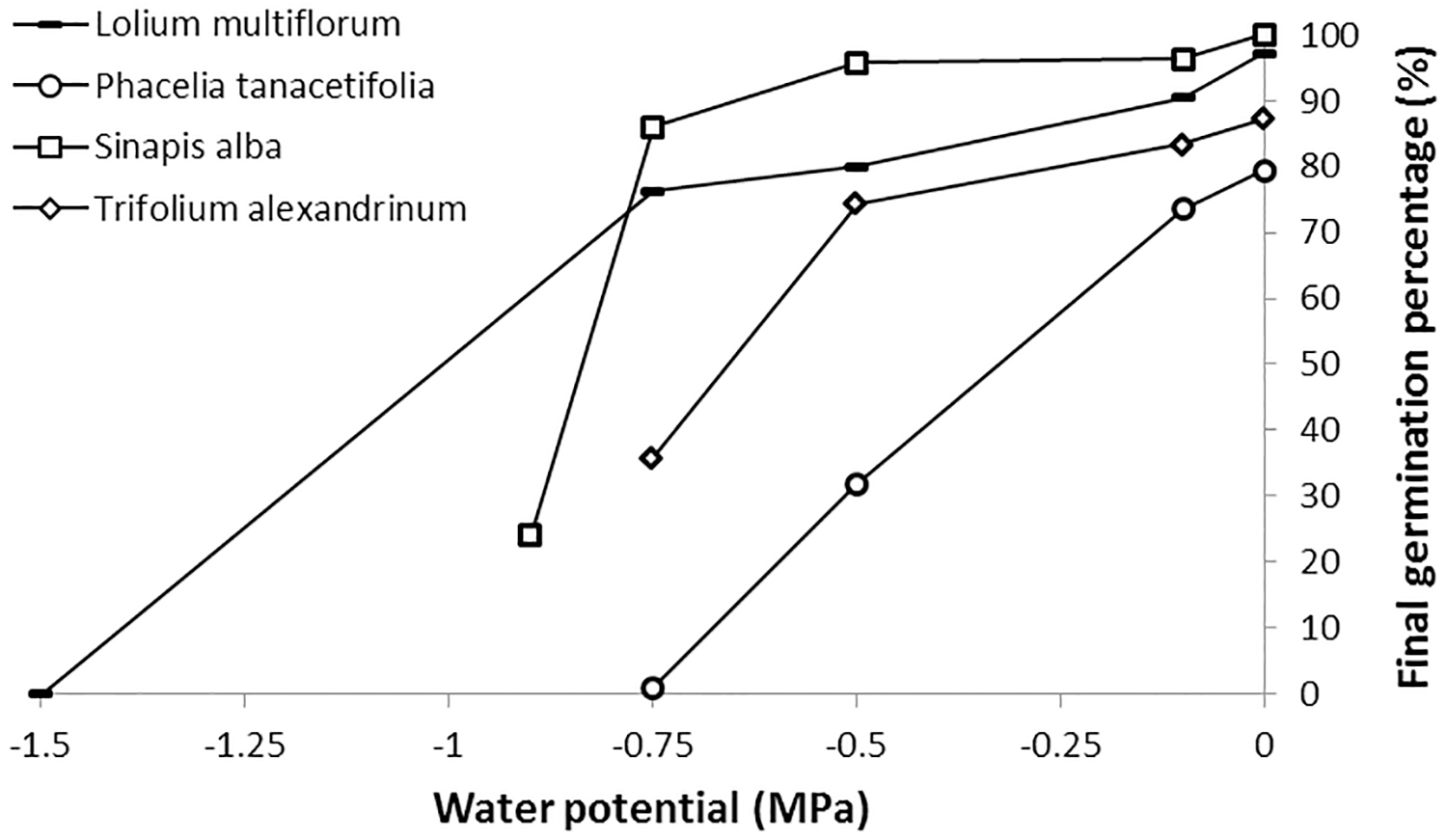
Examples of the influence of water potential (MPa) on final germination percentage of four species from various families.

**Fig 6:**
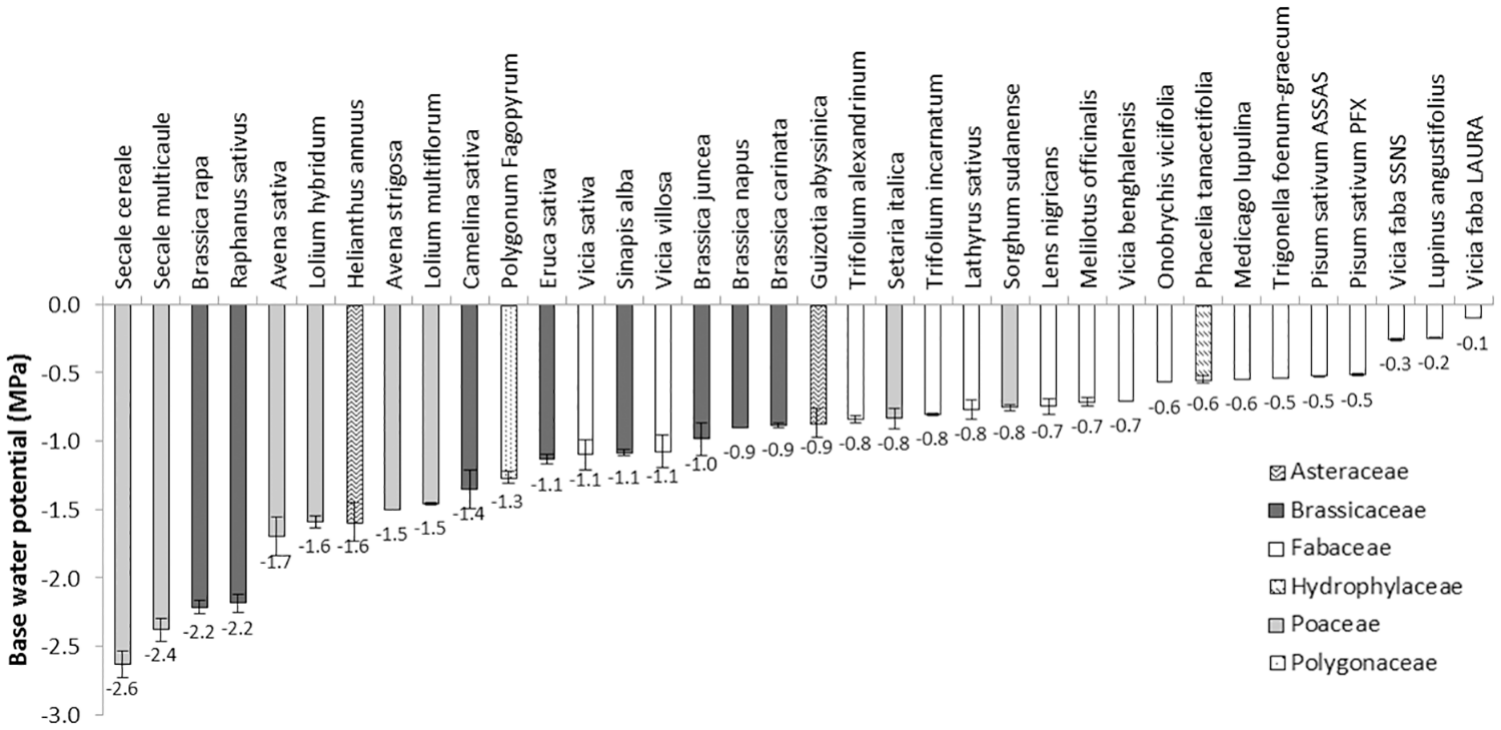
Base water potential for germination calculated for the 36 cover crops. Patterns and shades of gray correspond to botanical families. Data are the means of both 20^th^ and 30^th^ percentiles.

The final germination percentage of all cover crop species decreased with water potential (Fig 5). The germination rate also decreased with water potential (data not shown), which helped determine the base water potential. Ψ_b_ varied widely among species, from -0.1 MPa for *Vicia faba* var. LAURA (the species with the highest seed mass) to -2.6 MPa for *Secale cereale*, with a mean of -1.1 MPa for all cover crops (Fig 6). Mean base water potential by family was -1.3±0.5 MPa for Asteraceae, -1.4±0.6 MPa for Brassicaceae, -0.6±0.3 MPa for Fabaceae and -1.6±0.7 MPa for Poaceae. Overall, Poaceae (especially C_3_ species) and Brassicaceae had lower Ψ_b_ than Fabaceae, which were more sensitive to water potential for germination. Finally, no significant relationship (*P* = 0.6) was found between Ψ_b_ and T_b_; however, Ψ_b_ was positively and significantly (*P*<0.05) correlated with seed weight even though the R² was low (R² = 0.21).

### Species functional groups for germination

**Fig 7:**
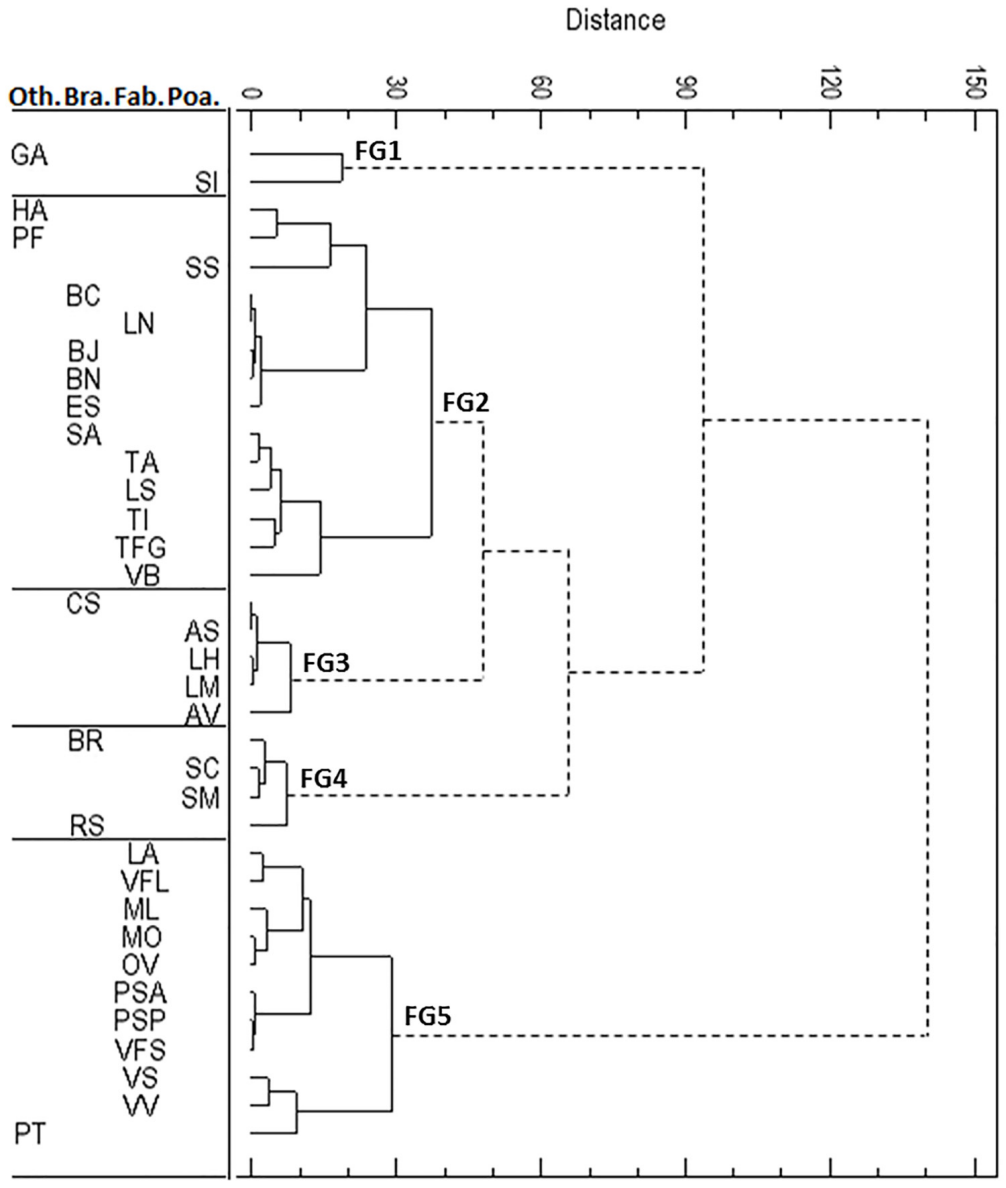
Hierarchical classification based on cardinal temperatures (T0, Tmax and Topt) and base water potential estimated on 36 cover crops using the Ward method (P<0.05). Solid lines represent distances between species of each of five functional groups (FG), and dashed lines represent distances between FGs. See Table 1 for the definition of species abbreviations; ‘Bra.’ is ‘Brassicaceae’, ‘Fab.’ is ‘Fabaceae’, ‘Poa.’ is ‘Poaceae’ and ‘Oth.’ is ‘Other families’.

The hierarchical classification allowed the cover crops to be classified into five functional groups that correspond to different germination profiles (Fig 7). All differences between groups were significant at *P*<0.0001.

Functional group 1 consisted of two species (*Guizotia abyssinica* and *Setaria italica*) with the highest T_opt_ and T_max_ but that could not germinate at low temperatures due to a significantly higher T_0_ than other groups (Table 2). Conversely, group 2 consisted of 14 species (39% of all species) from various families, mainly Brassicaceae and Fabaceae, that could germinate at relatively low temperatures (mean T_0_ = 1.3°C) and had high mean T_max_ (39.3°C) and mean T_opt_ (30.7°C). Group 3 consisted mainly of Poaceae and *Camelina sativa*, which had an intermediate ability to germinate at moderate mean T_opt_ (28.1°C) and T_max_ (35.0°C). Group 4, consisting of two Brassicaceae and the two *Secale* spp., had mean T_min_ and T_max_ that did not differ significantly from those of group 2 but a higher mean T_opt_, and they could germinate at a lower mean water potential than other groups. It also had a relatively lower mean Ψ_b_ than those of groups 1, 2 and 5. Finally, group 5 consisted of ten Fabaceae and *Phacelia tanacetifolia* and had the significantly lowest mean T_max_ and T_opt_.

**Table 2:** Mean (± 1 standard deviation) cardinal temperatures and base water potential of the species in each functional group defined from hierarchical classification.

Functional group	Minimal temperature	Optimal temperature	Maximal temperature	Base water potential
1	10.0 ± 1.8 b	32.4 ± 5.2 bc	41.2 ± 2.4 c	-0.9 ± 0.1 c
2	1.3 ± 1.6 a	30.7 ± 3.2 b	39.3 ± 2.3 c	-0.9 ± 0.3 c
3	0.4 ± 0.4 a	28.1 ± 1.7 ab	35.0 ± 1.4 b	-1.5 ± 0.1 b
4	0.4 ± 0.6 a	34.2 ± 2.2 c	38.6 ± 1.2 c	-2.4 ± 0.2 a
5	0.8 ± 0.7 a	24.9 ± 3.0 a	32.1 ± 2.2 a	-0.6 ± 0.3 c

Letters ‘a’, ‘b’, ‘c’ indicate significantly (P<0.05) different means.

## Discussion

This study provides results on the influence of two major environmental factors on germination—temperature and water potential—which influence the establishment of cover crops during the fallow period. Importantly, they are sown in summer, when soil temperature and water availability can vary greatly according to location and date of sowing.

Cardinal temperatures, T_b_ and Ψ_b_ were determined for a wide range of cover crop species. These data were not available in the literature for such a wide range of species and using the same method for all species, which enable them to be compared. Our results were generally consistent with those found in the literature, even though most T_b_ references were estimated for emergence (T_b-emerg_) measured in field experiments (Table 3). *Setaria italica* and *Polygonum fagopyrum* were distinguished from other species in this study for their high T_b_ (7.8°C and 10.6°C, respectively), which was consistent with literature values (T_b-emerg_ = 10.9°C and 11.1°C, respectively [27,28]). However, the literature reported different values for parameters of certain species, for example, T_b_ for *Onobrychis viciifolia* (5.5°C, vs. 0.0°C here) or Ψ_b_ for *Lupinus angustifolius* (-1.0 to -2.0 MPa, vs. -0.2 MPa here). These differences are probably due in part to differences in the methods, calculations and cultivars used. These differences could also be attributed to the age of seeds and/or nutrient status of the seeds, partly to possible effects of seed lots and more generally the quality of seeds.

**Table 3:** Cardinal temperatures for germination and emergence in the field (T_b-emerg_) from the literature for cover crop species in this study.

Species	T_b_ (°C)	T_opt_ (°C)	T_max_ (°C)	T_b-emerg_ (°C)	Ψ_b_ (MPa)	Reference
*Helianthus annuus*	1.0 to 5.1	34.0 to 36.7	45.5 to 50.9		-1.2	Mwale et al. 2003 [29]
				7.9		Angus et al. 1981 [27]
*Camelina sativa*				-0.7		Allen et al. 2014 [30]
*Brassica napus*	2.6 to 3.5	22 to 24				Marshall and Squire 1996 [31]
				2.6		Angus et al. 1981 [27]
*Sinapis alba*	3.3	27.0	39.0		-1.0	Dorsainvil 2002 [15]
*Avena sativa*				1.6		Yusoff et al. 2012 [32]
				2.2		Angus et al. 1981 [27]
*Setaria italica*				10.9		Angus et al. 1981 [27]
	9.3	37.0	46.0			Kamkar et al. 2006 [28]
*Lolium multiflorum*	4.6	27.0	38		-2.5	Dorsainvil 2002 [15]
	1.8					Yusoff et al. 2012 [32]
*Secale cereale*				2.6		Angus et al. 1981 [27]
*Faba bean*	0.40	25.4	37.08			Dumur et al. 1990 [33]
				1.2		Yusoff et al. 2012 [32]
				1.7		Iannucci et al. 2008 [34]
*Lupinus angustifolius*	0.0 to 3.0	20.0			-1 to -2	Dracup et al. 1993 [35]
*Pisum sativum*				1.4		Angus et al. 1981 [27]
	0.0	29.0	40.0			Olivier and Annandale 1998 [36]
	-0.4 to 9.6					Raveneau et al. 2011[37]
				2.4		Iannucci et al. 2008 [34]
*Onobrychis viciifolia*				5.5		Iannucci et al. 2008 [34]
*Trifolium alexandrinum*				3.5		Iannucci et al. 2008 [34]
*Vicia sativa*				0.0		Iannucci et al. 2008 [34]
*Vicia villosa*				1.9		Iannucci et al. 2008 [34]
*Polygonum fagopyrum*				11.1		Angus et al. 1981 [27]

Knowing the cardinal temperatures and Ψ_b_ values is useful for choosing cover crop species according to climatic conditions. In summer, soil temperature in the seedbed can be high; therefore, it is especially crucial to know the optimum and maximum temperatures and the Ψ_b_ for the germination of species. Most cover crop species evaluated in this study had a high optimal temperature and relatively low Ψ_b_. However, certain species, such as *Phacelia tanacetifolia* and *Vicia sativa*, did not germinate at temperatures > 30°C: these species should adapt better to sites with relatively mild temperatures. Most Fabaceae species were sensitive to low water availability, which indicates that they are better suited to rainy climates. All but a few species tested in this study reached an adequate final germination percentage within the optimal temperature range. The inability of some species to reach a high seed germination percentage is most likely due to the presence of hard seeds in the seedlot. This property seems specific and is well described for *Medigaco*, whose hard seed coats may lead to low germination [38].

The botanical families displayed a range of temperature and water-potential threshold values. Thus, family-related seed characteristics (e.g. structure, integument, composition) are not major drivers of the observed effects of temperature and water potential on germination. A species’ geographic origin has more influence on its ability to germinate, as reported by Dürr et al. (2015) [13] for a wide range of species from trees to crops. In our study, the functional group with the highest mean minimum and maximum temperature (group 1) contained two tropical species (*Setaria italica* and *Guizotia abyssinica*) from two different families (Poaceae and Asteraceae). Similarly, the C_4_ photosynthetic pathway, frequently found in tropical species, also seems to distinguish Poaceae species, since the two C_4_ species had higher cardinal temperatures than C_3_ species. We did not find a significant relationship between T_b_ and Ψ_b_ for all cover crop species combined, unlike Dürr et al. (2015) [13]. This could be because these species already represent a group of species from agrosystems that were domesticated by humans. This domestication could have decorrelated germination traits, as suggested for leaf functional traits for the same species [39]. It seems that species with high seed weight, such as *Vicia faba*, *Lupinus angustifolius* and *Pisum sativum*, tend to have a relatively high base water potential, maybe because they need a relatively large amount of water to achieve imbibition.

We organized and clarified the diverse germination responses through functional groups composed of species with the same range of cardinal temperatures and Ψ_b_ for germination and that thus would be adapted to the same climatic conditions. One direct application of these groups would be to help in choosing which cover crop to sow based on its ability to germinate under the climatic conditions of the site. For example, in southern France, where summer is often dry and hot, it would be better to sow a species from group 4, with a high optimal temperature and a low base water potential. Another application could be to help design cover crop mixtures when two or more species are sown and grown at the same time and under the same conditions. In this case, one way to benefit from these germination abilities would entail decreasing the climatic risk that decreases cover crop emergence by choosing several species with contrasting germination abilities (from different functional groups) to spread the risk of non-emergence. For example, intercropping *Guizotia abyssinica* (group 1), well adapted to high temperatures, with *Vicia sativa* (group 5), which prefers lower temperatures, and *Avena sativa* (group 3), in an intermediate position, could increase the chances of obtaining acceptable emergence and homogenous cover regardless of the seedbed temperature.

Species most adapted to the sowing conditions will germinate and emerge rapidly, which would help the crop establish itself and compete for resources. Species with early emergence in the field may also cover the soil earlier, protecting the soil sooner, and have faster root and shoot growth, which would capture nitrate sooner, decreasing nitrogen loss. This demonstrates the importance of adapting the choice of cover crop species to the climatic conditions at sowing to help provide ecosystem services during fallow periods. When a specific family is desired during fallow periods (e.g., Brassicaceae, for their effective rooting system and soil structuring properties), one can adapt species choice to the climatic conditions because of the variability in response of germination to temperature and water potential by species within the same family. The same kind of approach was proposed for species used in restoration programs of wild spaces [40].

To help select the most appropriate species to sow as a function of climate, date of sowing or different sowing conditions, T_b_, Ψ_b_ and the cardinal temperatures calculated in this study can be used in crop emergence models to simulate the emergence of cover crops under different pedoclimatic conditions. Several studies used dynamic models (e.g. SIMPLE, STICS) to study cover crop emergence influenced by climate and sowing date [8,9,41,42]. Accurately predicting the date of cover crop emergence is crucial, especially for simulating scenarios in which the emergence date has not been observed. For example, the STICS model [43,44] is a dynamic soil-plant model that can be used to study the influence of cover crops on the provision of short- and long-term ecosystem services [3,4,45,46]. Cover crop species from several botanical families can be simulated under a variety of soil characteristics, climate and crop management practices (e.g. sowing date, destruction date, crop residue management). When data are not available, cardinal temperatures for germination could be used in crop models as an initial estimate of the cardinal temperatures for plant growth and development. Parent and Tardieu (2012) [47] showed that the temperature response for germination rates and growth rates was similar for a variety of species. However as germination is only the first stage for plant establishment, further studies should provide complementary information on seedling growth after germination so as to have a more complete characterization of the different species and also to test the idea of common temperature response at different stages.

The concept of functional groups for germination could also be useful in modeling as a set of parameters to parametrize a germination profile or, when the species level is not required, a group of species. These groups reduce the diversity observed at the species level and increase the generality of the approach.

## Conclusion

We calculated cardinal temperatures, T_b_ and Ψ_b_ for the germination of a wide range of cover crop species for which temperature and water potential are crucial factors to consider at sowing. Our results showed that most of the species were adapted to summer sowing due to their relatively high optimal temperatures and low water potentials, although Fabaceae seemed more sensitive. Our data can also be useful as germination parameters in crop models to simulate the emergence of cover crops under different pedoclimatic conditions.

We grouped species into functional germination groups that classified species according to their sensitivity to temperature and water potential, independent of their botanical families. These groups are useful for adapting the choice of cover crop species to sow according to climatic conditions in order to favor adequate crop establishment and the provision of ecosystem services during the few months of the fallow period.
